# Feeding the Family—A Food Is Medicine Intervention: Preliminary Baseline Results of Clinical Data from Caregivers and Children

**DOI:** 10.3390/nu18020354

**Published:** 2026-01-22

**Authors:** Gabriela Drucker, Christa Mayfield, Elizabeth Anderson Steeves, Sara Maksi, Tabitha Underwood, Julie Brown, Marissa Frick, Alison Gustafson

**Affiliations:** 1 Department of Behavioral Science, College of Medicine, University of Kentucky, Lexington, KY 40506, USA; 2 Dietetics and Human Nutrition, Martin-Gatton College of Agriculture, Food, and Environment, University of Kentucky, Lexington, KY 40546, USA; christa.mayfield@uky.edu (C.M.); sara.maksi@uky.edu (S.M.); juliebrown@uky.edu (J.B.); marissa.frick@uky.edu (M.F.); alison.gustafson@uky.edu (A.G.); 3 Center Health Impact Nutrition, Omaha, NE 68022, USA; easteeves@centerfornutrition.org; 4 UofL Health, Louisville, KY 40202, USA; tabitha.underwood@uoflhealth.org

**Keywords:** food insecurity, social determinants of health, Food is Medicine

## Abstract

**Background/Objectives**: Food is Medicine (FIM) programs have been shown to be effective at addressing food and nutrition insecurity among individuals. However, more evidence is needed to determine effective interventions at the household level and their impact on child health outcomes. *Feeding the Family* is a randomized controlled trial which aims to determine whether the amount of food provided and the ability to select foods in FIM interventions have an incremental effect on child and caregiver clinical outcomes relative to nutrition counseling alone. The objective of this paper is to describe the population at baseline among those enrolled in *Feeding the Family*, an FIM family intervention. **Methods**: A pragmatic randomized controlled trial (pRCT) with a 2 × 2 factorial study design was used at an urban primary care clinic. Participants were randomized into one of four arms for a 3-month intervention: (1) medically tailored meals (MTMs), (2) grocery prescription (GP), (3) combined MTMs + GP, and (4) delayed control. Primary outcomes consisted of child and caregiver biomarkers (BMI, blood pressure, A1c, LDL, and HDL). Secondary outcomes included child and caregiver dietary behaviors, nutrition security, and food security. Spearman correlations and Kruskal–Wallis rank sum tests determined correlations between caregiver and child biomarkers, as well as correlations between caregiver socioeconomic factors and child outcomes, respectively. **Results**: Thirty-one caregivers and fifty-one children were enrolled. Nearly 90% of caregivers reported low–very low household food security; 93.6% experienced ongoing financial strain. Several caregiver–child biomarker correlations were observed, including caregiver and child BMI (r = 0.59, *p* = 0.043), caregiver LDL and child A1c (r = −0.79, *p* = 0.004), and caregiver total cholesterol and child BMI (r = −0.62, *p* = 0.032). In addition, food assistance status was associated with child vegetable intake (H = 6.16, *df* = 2, *p* = 0.046), and caregiver food security score was associated with child food security score (H = 18.31, *df* = 9, *p* = 0.032). **Conclusions**: There are robust correlations between caregiver and child clinical outcomes at baseline. These findings underscore the need for FIM research to examine how a tailored program can improve the clinical outcomes of entire households to address health disparities effectively.

## 1. Introduction

Food insecurity is defined as a household-level socioeconomic condition of limited or uncertain access to adequate food [1] and is a major social determinant of health [2]. Food insecurity has been strongly linked to worse diet quality, poorer mental health outcomes, and increased chronic disease burden, including higher rates of diabetes and heart disease [3,4,5,6]. For example, cardiovascular disease (CVD) and type 2 diabetes (T2DM) are more prevalent in food-insecure households [6,7,8,9]. Studies have shown that child diet quality and health outcomes such as obesity and CVD risk are negatively impacted by food insecurity as well [10,11,12]. Furthermore, low-income and racial/ethnic minority groups experience higher rates of food insecurity and diet-related chronic diseases [3,13,14]. Given this interaction between food insecurity, socioeconomic and racial/ethnic disparities, and poor health outcomes, there is an urgent need for effective clinic–community interventions to reach the populations that are experiencing the greatest burden.

Food is Medicine (FIM) programs provide an avenue for clinic-linked resources delivered by community-based organizations to address food insecurity. FIM interventions such as grocery prescription (GP) programs (i.e., prescription vouchers or cards that can be redeemed in exchange for healthy food at participating food vendors) and medically tailored meals (MTMs) (i.e., fully prepared meals delivered to participants on a weekly basis tailored to specific health needs and comorbidities) have been shown to be effective at improving health outcomes as standalone programs on the individual level [15,16]. However, recent studies have indicated a lack of change in clinical outcomes among adults participating in these programs [17]. Others point out a key concern that overemphasizing the effect of these programs can affect clinical outcomes at scale [18].

FIM interventions are being piloted to address diet-related chronic disease at an increasing rate [19]. However, much of the existing literature focuses on the type of food provision (i.e., medically tailored meals versus grocery/produce prescriptions) rather than the specific features of these programs that drive the most effective outcomes. While some programs have explored the intensity and duration of interventions, recognizing that the volume of food provided may influence dietary behavior and the household food environment, there is a key gap in rigorous research testing specific features such as dose of food, participant choice, and scaling by household [19,20]. However, few studies have systematically investigated these aspects of FIM intervention. Addressing these gaps is critical for understanding how to develop FIM interventions that optimize impact and scalability in households facing socioeconomic challenges.

To address gaps in the FIM literature, we conducted a pragmatic randomized control trial (pRCT) designed to address household food insecurity, with varying levels of food package size and food choice, in addition to supplemental nutrition and mental health counseling, to determine whether dose and choice can have an incremental effect on managing clinical health outcomes for both caregivers and children within a household relative to nutrition counseling alone. Establishing the baseline characteristics of study participants is essential for understanding the context in which the intervention is being implemented. The objective of this paper is to describe the population at baseline among those enrolled in *Feeding the Family*, a Food is Medicine family intervention. Our findings will provide insight into the population served and the program’s effectiveness in addressing food insecurity and improving health outcomes.

## 2. Materials and Methods

### 2.1. Study Design and Setting

The *Feeding the Family* intervention was a collaboration between the University of Kentucky (UK) and UofL Health. This study took place at UofL Physicians, Primary Care, Parkland, a large primary care clinic serving lower-resource adults in Louisville, Kentucky, where 13% of the population—and nearly 1 in 5 children—is food insecure [21]. In addition, approximately 7% and 13.5% of adults in Louisville have heart disease and diabetes, respectively [22]. As the sole outpatient site for this trial, Parkland’s patient population predominantly identifies as Black or African American, which is representative of the urban community. The study was a pragmatic randomized controlled trial (pRCT), using a 2 × 2 factorial design, and here, we provide a baseline analysis of the study sample. The protocol for this randomized controlled trial was approved by the University of Kentucky Institutional Review Board (IRB #96706). This study is part of a registered pragmatic randomized controlled trial (ClinicalTrials.gov Identifier: NCT06784310).

### 2.2. Eligibility Criteria

Participants were recruited from the outpatient UofL Health clinic from April to June 2025. Participants were from low-income families enrolled in Medicaid, with diet-related chronic conditions, household food insecurity, and children at greater risk of poor health outcomes. Adults were eligible if (1) they were between the ages of 18 and 64, (2) they had a clinical diagnosis of hypertension or type 2 diabetes mellitus (T2D), they (3) responded “yes” to at least one question on the Hunger Vital Sign^TM^ food insecurity screening tool [23], and (4) they had at least one child residing in the household. Additional inclusion criteria included English language fluency, no plans to move away from the area for at least one year, and the ability to participate in food delivery. Households were excluded if they did not have at least one child between the ages of 5 and 18 residing in the home at least 50% of the time or if they had already been enrolled in a weight or nutrition-related intervention in the past 12 months.

### 2.3. Recruitment, Consent, Assent, and Randomization

**Figure 1:**
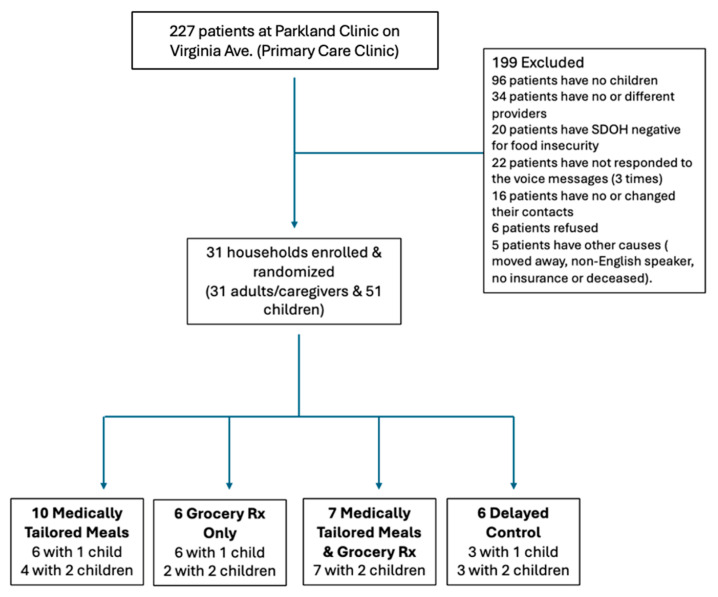
Feeding the family intervention study flow diagram.

Eligible participants were identified by the participating clinic, who identified eligible patients based on a medical record review. Patients were contacted by a UofL community health worker employed at the clinic who reached out via phone to assess their interest and obtain verbal permission to share their contact information, which was uploaded to a secure database that could be accessed by the research team. The research team contacted interested individuals to complete additional screening, obtain written informed consent, and document HIPAA authorization. For the caregiver, the informed consent was authorized via e-consent, which allowed for an electronic signature. The consent was read over the phone or sent via text for the participant to complete online. For child participants, consent was obtained from the legal guardian. To obtain assent from the child, a Zoom meeting was scheduled to ensure there was no coercion with regard to their participation. Assent was then verbally given at the Zoom meetings by each child in the household. Following enrollment, participants were randomized by household into one of four study arms. Randomization was not blinded and block randomization was utilized in light of the small sample size. Individuals were randomized to control or intervention and then randomized into one of three treatment arms throughout the study to prevent imbalance between the arms. The sample size was originally set at 60 caregivers for enrollment based on the UofL Health patient population for food-insecure adults with diagnosed hypertension (Figure 1).

### 2.4. Study Arms

A total of 227 patients were screened for eligibility at the Parkland clinic. Of these, 199 were excluded for reasons such as not having children (*n* = 96), the lack of a consistent provider (*n* = 34), screening negative for food insecurity (*n* = 20), or being unreachable/unwilling to participate. Overall, 31 households (comprising 31 adults/caregivers and 51 children) were enrolled and randomized across the four intervention arms: MTMs (*n* = 10), GP (*n* = 6), MTMs + GP (*n* = 7), and delayed control (*n* = 6). Each arm included families with one or two children.

The 2 × 2 factorial design included four interventions arms: medically tailored meals (MTMs), grocery prescription (GP), combined medically tailored meals + grocery prescription (MTMs + GP), and a delayed intervention control group.

#### 2.4.1. Medically Tailored Meals (MTMs)

In the MTMs arm, each household in this arm received 5 medically tailored meals per week for the caregiver and each enrolled child (up to two children) for 12 weeks. Meal plans were developed by registered dietitians and tailored to meet dietary needs based on specific health needs and clinical diagnoses. Meals are based on American Heart Association Dietary Approaches to Stop Hypertension (DASH) macronutrient profiles for fat, protein, carbohydrates, and sodium. Meals were provided through a local food bank and certified FIM provider in Louisville, KY, USA. Weekly delivery was facilitated through a contract with an online food delivery company.

#### 2.4.2. Grocery Prescription (GP)

Each household received monthly grocery prescription benefits valued at USD 50 per adult and USD 50 per child (up to two children) to be spent on eligible healthy food items. The USD 50 prescription covered approximately the same number of meals per person as provided in the MTMs arm. Grocery items were deemed eligible for purchase based on the American Heart Association Dietary Approaches to Stop Hypertension (DASH) macronutrient guidelines for fat, protein, and carbohydrates, with additional considerations for the micronutrient sodium. A list of approved food items (fruit, vegetables, low-fat dairy, and low-fat protein animal and vegetable protein sources) was developed with the assistance of a registered dietitian. The GP allows for integration between allowable food items within the shopping platform. Grocery prescriptions were provided to households in either of the following two formats:

1. In-store option: participants received a food benefit card—a debit-style card used by recipients to purchase eligible healthy grocery items—to be used at participating grocery stores (a supermarket retailer chain with 25 store locations across Louisville).
2. Online option: participants accessed grocery benefits through an online grocery delivery platform, which allowed for home delivery of eligible healthy grocery items.

#### 2.4.3. Combined MTMs + GP

The caregiver received MTMs while each child received USD 50 in grocery prescription benefits (available for up to two children).

#### 2.4.4. Delayed Control

Participants received standard care and were offered the intervention following completion of follow-up data collection.

### 2.5. Measures

Primary outcomes of interest include child and caregiver/adult BMI percentile, blood pressure, hemoglobin A1c, and lipid panels. The referral coordinator at UofL collected both the adult and child’s BMI and blood pressure from their electronic health record (EHR) via the Epic system. The child’s BMI percentile was automatically calculated in the EHR from height and weight using age- and gender-specific CDC growth charts [24]. The bloodwork was ordered as part of standard care in the clinic for the management of patients who have overweight or obese BMI percentiles. The bloodwork ordered by physicians included hemoglobin A1c, total cholesterol, high-density lipoprotein (HDL), and low-density lipoprotein (LDL), and the tests were conducted by trained phlebotomists on-site at the clinic or at affiliated diagnostic centers.

Secondary outcomes of interest included parent self-reported child and parental dietary behavioral outcomes (child fruit and vegetable intake/parent fruit and vegetable intake), family stress model, nutrition security, and food security. Child and parent fruit and vegetable intake were measured using items adapted from the Behavioral Risk Factor Surveillance System (BRFSS) Fruit and Vegetables Section [25], which assesses frequency-based consumption of common foods. Family stress was assessed using validated measures including the Patient Health Questionnaire for Depression and Anxiety [26], a four-question screener used to detect the two disorders, and the Perceived Stress Scale [27], a precise measure of personal stress. Food security was measured using the 18-item US Household Food Security Survey Module (HFSSM) [28], a validated tool assessing household-level access to adequate food over the past 12 months, and the Health Vital Sign [23]—a two-question subset of the module. Nutrition security was defined by consistent access, availability, and affordability of foods that promote well-being and prevent or treat disease, evaluated through caregiver self-report on dietary quality and barriers to healthy eating.

### 2.6. Statistical Analysis

All study data—including participant screening, informed consent documentation, baseline and follow-up survey administration, and storage of clinical data—were collected and managed using REDCap [29]. Prior to analysis, the dataset was reviewed for accuracy and completeness. Data cleaning included checks for outliers and missing values. The analyses examined participant demographics, food and nutrition security status, dietary behaviors, and clinical risk profiles among both children and adults. In addition, preliminary correlations were explored between key exposures and baseline outcomes in order to better understand the context in which intervention effects will be assessed and generate hypotheses about potential mediators or moderators. For families with more than one child, only data from Child 1 was analyzed, since all families had at least one child.

Descriptive statistics were conducted to characterize the sample at baseline, including frequencies and proportions for categorical variables and means or medians with standard deviations for continuous variables, as appropriate. Nonparametric statistical tests were used due to non-normal distributions observed in several outcome variables. Spearman’s rank correlation coefficients were calculated to assess correlations between caregiver and child biomarkers. Kruskal–Wallis rank sum tests were used to examine differences in child outcomes across categories of caregiver-level socioeconomic and behavioral factors. All analyses were conducted using R, version 3.6.2 [30].

## 3. Results

### 3.1. Baseline Characteristics

Table 1 provides a description of the study sample. A total of 31 caregivers and 51 children were enrolled in the study. Among caregivers, the majority were female (80.4%) and identified as Black or African American (93.5%). Most caregivers reported annual household incomes under USD 10,000, with 58.1% of households earning less than USD 10,000 per year. When reporting on employment status, 35.5% of caregivers responded that they were employed for wages, and 19.4% reported that they were unable to work due to disability. In terms of education, 51% had completed some college or a vocational degree. The average household size was 3.77 individuals (SD = 1.20). A majority of caregivers (72%) reported utilizing Supplemental Nutrition Assistance Program (SNAP) benefits; 71% participated in Medicaid, and a majority reported experiencing financial strain sometimes or rarely (67.7%) in their daily lives. At baseline, the majority of households experienced food insecurity. At the household level, *N* = 19 household or 61.3% reported very low food security based on the USDA Food Security Scale [28]. When asked these questions at the individual level, *N* = 19 or 58.1% of caregivers reported that their own food security was very low. At the child level, 48.4% of caregivers reported having low food security for their child(ren) and 25.8% reported having very low food security for their child(ren). Only six households (19.4%) had children with a high level of food security.

### 3.2. Parent–Child Biomarker Correlations

Several notable correlations between caregiver and child biomarkers were observed at baseline. Caregiver and child BMI were moderately correlated (r = 0.59, *p* = 0.043); thus caregivers with a high BMI had children in the household who also had a high BMI. This result is not unique, but points to the key need to target interventions at both the caregiver and child at the same time to improve healthy eating patterns and practices. Caregiver hemoglobin A1c was positively correlated with child triglycerides (r = 0.60, *p* = 0.052). These biomarker correlations suggest that not only does weight correlate, but metabolic health outcomes from a young age are correlated with caregiver markers. Thus, it is not enough to intervene in healthy eating; rather, a targeted approach is needed to address dietary intake to improve these specific clinical biomarkers. A strong negative correlation was observed between caregiver LDL and child hemoglobin A1c (r = −0.79, *p* = 0.004). When LDL is lower, childhood hemoglobin A1C is also lower, confirming previous hypotheses about the impact that caregiver health outcomes have in relation to childhood outcomes. Caregiver total cholesterol showed a significant negative correlation with child BMI (r = −0.62, *p* = 0.032) (Table 2).

### 3.3. Correlations Between Caregiver Socioeconomic Factors and Child Outcomes

Correlations between several caregiver-level socioeconomic variables and child dietary intake, as well as food security outcomes, were examined. Caregiver food assistance status (categorized as receiving support from programs such as SNAP, WIC, food pantries, or no food assistance) was significantly associated with child vegetable intake, such that those who reported receiving government nutrition assistance had a higher intake of vegetables compared to those who did not receive assistance (H = 6.16, *df* = 2, *p* = 0.046). These results informed this pilot study, which aimed to examine how SNAP benefits and other government nutrition assistance programs can be the backbone of a Food is Medicine program while providing additional benefits to improve clinical outcomes (Table 3).

Caregiver total food security score was significantly associated with both child food security score (H = 18.31, *df* = 9, *p* = 0.032) and with categorical child food security status (e.g., high/marginal, low, or very low) (H = 18.43, *df* = 9, *p* = 0.030), such that those with higher food security status as a caregiver had children in their household with high food security status. These baseline pilot results point to the role of addressing caregiver and child food security status at the same time, since there is a potential interactive effect in the household (Table 3).

## 4. Discussion

The pilot study and our baseline presentation confirm our approach in this clinical trial, referred to as the *Feeding the Family* intervention study, to intervene with both the caregiver and children. Our study sample was predominantly Black, with high levels of financial strain and low or very low food security status. Our pilot study baseline results point to a strong correlation between biomarkers between caregivers and children in the household.

These findings suggest a potential correlation of metabolic risk factors between caregivers and children within a household. While other studies have pointed out similar findings [37,38,39,40], there is still limited understanding of how the mechanisms of food insecurity, nutrition security, and obesity may impact children [41]. These correlational findings point to the need to further examine how interventions targeting food insecurity and clinical outcomes are necessary for both caregivers and children [42].

Additionally, financial strain and participation in food assistance programs were associated with children’s dietary intake and food security status. These findings suggest that caregiver-level structural and economic conditions may influence children’s diet quality and food access at baseline, even prior to intervention delivery. These findings are consistent with the literature, which has shown that household factors contribute to dietary quality and food security in low-income households [43]. Overall, these findings underscore the impact of the social determinants of health in this population. By examining caregiver–child dyads, these findings emphasize that food security interventions should extend to the entire household to address health disparities effectively.

The *Feeding the Family* intervention provides a potential solution to addressing whole-family nutrition security through medically tailored meals, grocery prescription benefits, or a combination of both. To date, several small pilot studies have examined how Food is Medicine interventions can improve dietary intake and, potentially, clinical outcomes [16,44,45,46,47,48,49]. However, there are still many key aspects of implementing the Food is Medicine approach that have yet to be addressed. Uncovering the complex content and delivery systems involved in Food is Medicine programs is vital to help inform standards of care and provide support for potentially offering this service as a medically covered benefit [7,15,19,50]. Understanding how participants engage with and benefit from different arms of the study will be important in determining which interventions are effective at addressing household food security and improving long-term health outcomes. While there have been a limited number of studies investigating FIM interventions at the household level [17], other articles specifically note a lack of interventions that scale by household size, address multiple family members, or consider child involvement [49,51]. The identification of these gaps in the literature highlights the need for additional investigation in this relatively under-explored area of FIM. Overall, tailoring Food is Medicine programs to account for household size, preferences, and child-specific health needs may improve both engagement and effectiveness.

This analysis is strengthened by its use of multilevel data sources—including EHR-derived clinical biomarkers, validated food security measures, and caregiver-reported household demographics. The study was conducted in partnership with a UofL Health clinical site that serves historically marginalized communities, making this research especially relevant to this vulnerable population. In addition, our pilot feasibility study informs how future clinical and community partnerships may need to collaborate when running these programs outside of the research space [52]. Finally, by enrolling both caregivers and children, the dataset provides insight to explore intra-household patterns that are often overlooked in adult-only or child-only interventions.

Despite these strengths, the following limitations should be considered. First, the sample size was modest (*n* = 31 households), limiting statistical power and precluding subgroup analyses at this stage. In addition, because the clinic primarily serves adult patients, many enrolled child participants were not under the direct care of the providers at the recruitment site, which posed additional challenges when it came to collecting child-level clinical data. While demographic homogeneity (i.e., majority Black, low-income, English-speaking) supports focused equity-driven intervention design, it may limit generalizability to other populations or geographic regions. The analyses were limited by sample size, and thus stratification of key variables was not possible due to the small cell size. This was a feasibility pilot study, and the design was not powered for mediation analyses. Finally, data on dietary intake were provided by caregivers and may be subject to recall or social desirability bias.

These findings set the stage for examining the impact of the *Feeding the Family* intervention across multiple dimensions of health and well-being. Future analyses will explore how intervention assignment (e.g., MTMs, GP, or both) influences changes in child and adult clinical outcomes, dietary behaviors, and food security status. Given the observed caregiver–child biomarker correlations, it will be important to examine whether the intervention effects are mediated through improvements in household food security, reduced financial strain, or changes in dietary intake. Moderation analyses may also explore how baseline factors—such as household size, SNAP participation, or education—shape the effectiveness of different intervention arms. These data will also inform program implementation, such as which features of the intervention are most effective, feasible, and scalable for families managing chronic conditions in low-resourced settings.

## 5. Conclusions

This baseline descriptive report for a pilot feasibility study highlights the intersection of food insecurity, financial hardship, and chronic disease risk among urban, Medicaid-enrolled families. The results of our analyses point to key aspects of intervention development and design—specifically the need to engage with the entire household in a Food is Medicine program rather than just one sub-population. Given the correlations between biomarkers, in order to move the needle on health outcomes, both the caregiver and their children will need to be supported by Food is Medicine programs based on their needs and preferences. In addition, given the limited sample size, further large-scale randomized control trials are necessary to disentangle key aspects of food intervention content, such as dose, duration, and intensity while also balancing user preferences, needs, and constraints. The *Feeding the Family* trial is uniquely positioned to provide insight into some of these challenges by delivering tailored, multilevel support to both caregivers and children. These findings provide a critical foundation for evaluating the intervention’s impact.

## Figures and Tables

**Table 1:** Sociodemographic characteristics of enrolled households at baseline (*N* = 31).

	Caregiver	Child 1	Child 2
	*n* (%)	*n* (%)	*n* (%)
**Gender**			
Male	6 (19.4)	11 (35.5)	13 (65)
Female	25 (80.4)	20 (64.5)	7 (35)
**Race**			
Black of African American	29 (93.5)	27 (87)	20 (100)
More than one race	1 (3.2)	1 (0)	0 (0)
Prefer not to answer	1 (3.2)	1 (0)	0 (0)
**Annual Household Income**			
<USD 10,000	18 (58.1)		
USD 10,000–USD 34,999	8 (25.8)		
USD 35,000–USD 50,000	5 (16.1)		
**Employment Status**			
Employed for wages	11 (35.5)		
Self-employed	2 (6.5)		
Out of work	8 (25.8)		
Unable to work (disabled)	6 (19.4)		
**Education Level**			
9th–12th grade; no diploma	3 (9.7)		
High school grad or GED	9 (29.0)		
Completed vocational, trade, or some college	16 (51.6)		
Some college, no degree	11 (35.5)		
Bachelor’s degree or higher	3 (9.6)		
**Household Size**			
Average	3.77 (1.2 SD)		
**Nutrition Assistance**			
SNAP	23 (74.2)		
WIC	2 (6.5)		
**Government Assistance**			
CHIP	1 (3.2)		
TANF	3 (9.7)		
Medicaid	22 (71.0)		
None of the above	6 (19.4)		
Prefer not to answer	2 (6.5)		
**Financial Strain**			
Sometimes or rarely	21 (67.7)		
Often or always	10 (32.2)		
**Food Security Level**	**Household**	**Adult**	**Child 1**
High	0 (0)	2 (6.5)	6 (19.4)
Marginal	1 (3.2)	3 (9.7)	
Low	9 (29.0)	8 (25.8)	15 (48.4)
Very Low	19 (61.3)	18 (58.1)	8 (25.8)
Incomplete	2 (6.5)	0 (0)	2 (6.5)

SNAP—Supplemental Nutrition Assistance Program. WIC—Special Supplemental Nutrition Program for Women, Infants, & Children. CHIP—Children’s Health Insurance Program. TANF—Temporary Assistance for Needy Families. Food security categories follow USDA classification: high, marginal, low, and very low. Food security categories for children follows USDA classification: high/marginal, low, very low.

**Table 2:** Correlations between caregiver and child biomarkers.

		Child 1															
		BMI		BP (Systolic)		BP (Diastolic)		A1c		Cholesterol		HDL		LDL		Triglycerides	
		r	*p*	r	*p*	r	*p*	r	*p*	r	*p*	r	*p*	r	*p*	r	*p*
**Caregiver**	**BMI**	**0.59**	**0.042**	0.35	0.269	−0.003	0.991	0.27	0.391	−0.37	0.240	−0.40	0.195	−0.4	0.6	0.31	0.319
	**BP** (systolic)	0.44	0.147	−0.31	0.323	−0.41	0.189	0.18	0.577	−0.45	0.144	−0.30	0.350	0	1	−0.09	0.779
	**BP** (diastolic)	0.44	0.156	0.06	0.857	−0.27	0.391	0.36	0.256	−0.06	0.861	0.35	0.259	−0.8	0.2	−0.20	0.538
	**A1c**	0.20	0.548	0.30	0.375	−0.27	0.414	0.40	0.227	−0.08	0.815	−0.46	0.155	0.11	0.895	**0.60**	**0.052**
	**Cholesterol**	**−0.62**	**0.032**	−0.05	0.880	0.19	0.555	−0.40	0.201	0.55	0.065	0.46	0.135	0.8	0.2	−0.09	0.770
	**HDL**	−0.07	0.837	0.07	0.820	0.19	0.562	0.16	0.620	0.33	0.290	0.55	0.061	−1	0	−0.35	0.269
	**LDL**	−0.34	0.304	−0.22	0.509	0.32	0.331	**−0.79**	**0.004**	0.26	0.432	−0.21	0.526	1	0	0.05	0.894
	**Triglycerides**	−0.12	0.704	0.45	0.144	−0.12	0.720	0.11	0.723	−0.11	0.729	−0.43	0.159	0.8	0.2	0.66	0.018

Statistical tests used include Spearman’s rank-order correlation for ordinal data. Bolded *p*-values indicate statistical significance (*p* < 0.05). All analyses were conducted using R (version 3.6.2). BMI: body mass index (healthy child BMI 5th to <85th percentile [24]; normal adult BMI 18.5–24.9 [31]). BP: blood pressure (healthy child BP < 90th percentile [32]; normal adult BP < 120/<80 [33]). A1c: Hemoglobin A1c (normal HbA1c < 5.7% for both children and adults [34]). Cholesterol (acceptable child cholesterol < 170 [35], acceptable adult cholesterol < 200 [36]). HDL: high-density lipoprotein (acceptable child HDL ≥ 40 [35], protective adult HDL ≥ 60 [36]). LDL: low-density lipoprotein (acceptable child LDL < 110 [35], acceptable adult LDL < 100 [36]). Triglycerides (acceptable child triglycerides < 90 [35], normal adult triglycerides < 150 [36]).

**Table 3:** Correlations between caregiver sociodemographic factors and child outcomes.

	Child 1																	
	Juice Intake			Fruit Intake			Leafy Greens			Vegetables			Food Security Score			Food Security Category		
Caregiver	H	*df*	*p*	H	*df*	*p*	H	*df*	*p*	H	*df*	*p*	H	*df*	*p*	H	*df*	*p*
**Household Income**	0.19995	4	0.9953	0.24907	4	0.9929	7.679	4	0.1041	0.12424	4	0.9981	3.2306	4	0.52	1.9964	4	0.7364
**Education Level**	6.9724	6	0.3234	5.0808	6	0.5335	4.1591	6	0.6552	6.7935	6	0.3404	6.7293	6	0.3466	1.8793	6	0.9305
**Financial Strain**	6.4495	3	0.09168	5.6793	3	0.1283	3.1363	3	0.3711	5.7779	3	0.1229	2.2733	3	0.5176	0.8331	3	0.8415
**Food Assistance**	1.6865	2	0.4303	5.4971	2	0.06402	1.3795	2	0.5017	**6.1642**	**2**	**0.04586**	2.5604	2	0.278	0.66318	2	0.7178
**Government Assistance**	1.3435	2	0.5108	1.4885	2	0.4751	0.94317	2	0.624	3.6653	2	0.16	0.50013	2	0.7787	0.023248	2	0.9884
**Food Security Score**	7.5738	9	0.5776	7.832	9	0.5512	16.012	9	0.06664	8.9647	9	0.4405	**18.307**	**9**	**0.03177**	**18.436**	**9**	**0.03044**
**Food Security Category**	2.0954	3	0.5529	1.5489	3	0.671	5.0356	3	0.1692	2.3207	3	0.5086	**16.065**	**3**	**0.0011**	**17.046**	**3**	**0.0006916**

The statistical tests used include Kruskal–Wallis H test for nonparametric group comparisons. Bolded *p*-values indicate statistical significance (*p* < 0.05). All analyses were conducted using R (version 3.6.2).

## Data Availability

The original contributions presented in this study are included in the article. Further inquiries can be directed to the corresponding author.

## Abbreviations

The following abbreviations are used in this manuscript:

FIM	Food is Medicine.
pRCT	Pragmatic randomized controlled trial.
MTMs	Medically Tailored Meals.
GP	Grocery Prescription.
BMI	Body Mass Index.
A1c	Hemoglobin A1c.
LDL	Low-Density Lipoprotein.
HDL	High-Density Lipoprotein.
CVD	Cardiovascular Disease.
T2DM	Type 2 Diabetes Mellitus.
UofL	University of Louisville.
DASH	Dietary Approaches to Stop Hypertension.
EHR	Electronic Health Records.
SNAP	Supplemental Nutrition Assistance Program.
WIC	Special Supplemental Nutrition Program for Women, Infants, and Children.
CHIP	Children’s Health Insurance Program.
TANF	Temporary Assistance for Needy Families.

## References

[1] Overview. USDA ERS—Food Security in the U.S. https://www.ers.usda.gov/topics/food-nutrition-assistance/food-security-in-the-us/definitions-of-food-security/.

[2] U.S. Department of Health and Human Services, Office of Disease Prevention and Health Promotion *Food Insecurity*. Healthy People 2030. https://odphp.health.gov/healthypeople/priority-areas/social-determinants-health/literature-summaries/food-insecurity#cit1.

[3] Coleman-Jensen A., Rabbitt M.P., Gregory C.A., Singh A. Household Food Security in the United States in 2021, ERR-309, US Department of Agriculture. https://www.ers.usda.gov/webdocs/publications/104656/err-309.pdf.

[4] Gundersen C., Ziliak J.P. (2015). Food Insecurity and Health Outcomes. Health Aff..

[5] Seligman H.K., Laraia B.A., Kushel M.B. (2010). Food insecurity is associated with chronic disease among low-income NHANES participants. J. Nutr..

[6] Thomas M.K., Lammert L.J., Beverly E.A. (2021). Food Insecurity and its Impact on Body Weight, Type 2 Diabetes, Cardiovascular Disease, and Mental Health. Curr. Cardiovasc. Risk Rep..

[7] Chang R., Javed Z., Taha M., Yahya T., Valero-Elizondo J., Brandt E.J., Cainzos-Achirica M., Mahajan S., Ali H.-J., Nasir K. (2022). Food insecurity and cardiovascular disease: Current trends and future directions. Am. J. Prev. Cardiol..

[8] Liu Y., Eicher-Miller H.A. (2021). Food Insecurity and Cardiovascular Disease Risk. Curr. Atheroscler. Rep..

[9] Casagrande S.S., Bullard K.M., Siegel K.R., Lawrence J.M. (2022). Food insecurity, diet quality, and suboptimal diabetes management among US adults with diabetes. BMJ Open Diabetes Res. Care.

[10] Fram M.S., Ritchie L.D., Rosen N., Frongillo E.A. (2015). Child experience of food insecurity is associated with child diet and physical activity. J. Nutr..

[11] South A.M., Palakshappa D., Brown C.L. (2019). Relationship between food insecurity and high blood pressure in a national sample of children and adolescents. Pediatr. Nephrol..

[12] Hammad N.M., Wolfson J.A., de Ferranti S.D., Willett W.C., Leung C.W. (2024). Food Insecurity and Ideal Cardiovascular Health Risk Factors Among US Adolescents. J. Am. Heart Assoc..

[13] Agurs-Collins T., Alvidrez J., ElShourbagy Ferreira S., Evans M., Gibbs K., Kowtha B., Pratt C., Reedy J., Shams-White M., Brown A.G.M. (2024). Perspective: Nutrition Health Disparities Framework: A Model to Advance Health Equity. Adv. Nutr..

[14] Morales D.X., Morales S.A., Beltran T.F. (2021). Racial/Ethnic Disparities in Household Food Insecurity During the COVID-19 Pandemic: A Nationally Representative Study. J. Racial Ethn. Health Disparities.

[15] Mozaffarian D., Aspry K.E., Garfield K., Kris-Etherton P., Seligman H., Velarde G.P., Williams K., Yang E. (2024). ACC Prevention of Cardiovascular Disease Section Nutrition; Lifestyle Working Group and Disparities of Care Working Group. “Food Is Medicine” Strategies for Nutrition Security and Cardiometabolic Health Equity: JACC State-of-the-Art Review. J. Am. Coll. Cardiol..

[16] Hager K., Du M., Li Z., Mozaffarian D., Chui K., Shi P., Ling B., Cash S.B., Folta S.C., Zhang F.F. (2023). Impact of Produce Prescriptions on Diet, Food Security, and Cardiometabolic Health Outcomes: A Multisite Evaluation of 9 Produce Prescription Programs in the United States. Circ. Cardiovasc. Qual. Outcomes.

[17] Doyle J., Alsan M., Skelley N., Lu Y., Cawley J. (2024). Effect of an Intensive Food-as-Medicine Program on Health and Health Care Use: A Randomized Clinical Trial. JAMA Intern. Med..

[18] Moran A.J., Roberto C.A. (2023). A “Food Is Medicine” Approach to Disease Prevention: Limitations and Alternatives. JAMA.

[19] Downer S., Berkowitz S.A., Harlan T.S., Olstad D.L., Mozaffarian D. (2020). Food is medicine: Actions to integrate food and nutrition into healthcare. BMJ.

[20] Gupta P., Karpman M., Waxman E., Allen E., Gonzalez D., Hinojosa S., Kennedy N. (2025). Public Perceptions of ‘Food Is Medicine’ Programs and Implications for Policy: Insights from the Well-Being and Basic Needs Survey and Qualitative Interviews.

[21] (2021). Food Security in Louisville. Greater Louisville Project. https://greaterlouisvilleproject.org/food-security/.

[22] UofL Health (2025). 2026–2028 Community Health Needs Assessment, Jefferson County.

[23] Hager E.R., Quigg A.M., Black M.M., Coleman S.M., Heeren T., Rose-Jacobs R., Cook J.T., de Cuba S.A.E., Casey P.H., Chilton M. (2010). Development and validity of a 2-item screen to identify families at risk for food insecurity. Pediatrics.

[24] Centers for Disease Control and Prevention (2022). Extended BMI-for-Age (5 to 19 Years).

[25] Centers for Disease Control and Prevention (CDC) (2017). Behavioral Risk Factor Surveillance System Survey Questionnaire.

[26] Kroenke K., Spitzer R.L., Williams J.B., Lowe B. (2009). An ultra-brief screening scale for anxiety and depression: The PHQ-4. Psychosomatics.

[27] Cohen S., Kamarck T., Mermelstein R. (1983). A global measure of perceived stress. J. Health Soc. Behav..

[28] U.S. Department of Agriculture, Economic Research Service Survey Tools: U.S. Household Food Security Survey Module (18-Item).

[29] Harris P.A., Taylor R., Thielke R., Payne J., Gonzalez N., Conde J.G. (2009). Research electronic data capture (REDCap)—A metadata-driven methodology and workflow process for providing translational research informatics support. J. Biomed. Inform..

[30] R Core Team (2019). R: A Language and Environment for Statistical Computing.

[31] National Heart, Lung, and Blood Institute (2025). Classification of Overweight and Obesity by BMI, Waist Circumference, and Associated Disease Risks.

[32] Flynn J.T., Kaelber D.C., Baker-Smith C.M., Blowey D., Carroll A.E., Daniels S.R., de Ferranti S.D., Dionne J.M., Falkner B., Flinn S.K. (2017). Clinical Practice Guideline for Screening and Management of High Blood Pressure in Children and Adolescents. Pediatrics.

[33] Whelton P.K., Carey R.M., Aronow W.S., Casey D.E., Collins K.J., Himmelfarb C.D., DePalma S.M., Gidding S., Jamerson K.A., Jones D.W. (2018). 2017 ACC/AHA/AAPA/ABC/ACPM/AGS/APhA/ASH/ASPC/NMA/PCNA Guideline for the Prevention, Detection, Evaluation, and Management of High Blood Pressure in Adults: Executive Summary: A Report of the American College of Cardiology/American Heart Association Task Force on Clinical Practice Guidelines. Hypertension.

[34] American Diabetes Association Professional Practice Committee (2024). 2. Diagnosis and Classification of Diabetes: Standards of Care in Diabetes-2024. Diabetes Care.

[35] (2011). Expert Panel on Integrated Guidelines for Cardiovascular Health and Risk Reduction in Children and Adolescents. Expert panel on integrated guidelines for cardiovascular health and risk reduction in children and adolescents: Summary report. Pediatrics.

[36] Grundy S.M., Stone N.J., Bailey A.L., Birtcher K.K., Blumenthal R.S., Braun L.T., de Ferranti S., Faiella-Tommasino J., Forman D.E., Goldberg R. (2019). 2018 AHA/ACC/Multi-Society Guideline on the Management of Blood Cholesterol. J. Am. Coll. Cardiol..

[37] Fabiani E., Strand M.F., Lindberg M., Goswami N., Fredriksen P.M. (2022). Variation in Child Serum Cholesterol and Prevalence of Familiar Hypercholesterolemia: The Health Oriented Pedagogical Project (HOPP). Glob. Pediatr. Health.

[38] Raitakari O.T., Juonala M., Kahonen M., Taittonen L., Laitinen T., Mäki-Torkko N., Järvisalo M.J., Uhari M., Jokinen E., Rönnemaa T. (2003). Cardiovascular risk factors in childhood and carotid artery intima-media thickness in adulthood: The Cardiovascular Risk in Young Finns Study. JAMA.

[39] Simmonds M., Burch J., Llewellyn A., Griffiths C., Yang H., Owen C., Duffy S., Woolacott N. (2015). The use of measures of obesity in childhood for predicting obesity and the development of obesity-related diseases in adulthood: A systematic review and meta-analysis. Health Technol. Assess..

[40] Acharya K., Feese M., Franklin F., Kabagambe E.K. (2011). Body mass index and dietary intake among Head Start children and caregivers. J. Am. Diet Assoc..

[41] Kral T.V.E., Chittams J., Moore R.H. (2017). Relationship between food insecurity, child weight status, and parent-reported child eating and snacking behaviors. J. Spec. Pediatr. Nurs..

[42] Seligman H.K., Angell S.Y., Berkowitz S.A., Elkind M.S.V., Hager K., Moise N., Posner H., Muse J., Odoms-Young A., Ridberg R. (2025). A Systematic Review of “Food Is Medicine” Randomized Controlled Trials for Noncommunicable Disease in the United States: A Scientific Statement from the American Heart Association. Circulation.

[43] Eicher-Miller H.A., Graves L., McGowan B., Mayfield B.J., Connolly B.A., Stevens W., Abbott A. (2023). A Scoping Review of Household Factors Contributing to Dietary Quality and Food Security in Low-Income Households with School-Age Children in the United States. Adv. Nutr..

[44] Berkowitz S.A., Delahanty L.M., Terranova J., Steiner B., Ruazol M.P., Singh R., Shahid N.N., Wexler D.J. (2019). Medically Tailored Meal Delivery for Diabetes Patients with Food Insecurity: A Randomized Cross-over Trial. J. Gen. Intern. Med..

[45] Lyonnais M.J., Kaur A.P., Rafferty A.P., Johnson N.S., Jilcott Pitts S. (2022). A Mixed-Methods Examination of the Impact of the Partnerships to Improve Community Health Produce Prescription Initiative in Northeastern North Carolina. J. Public Health Manag. Pract..

[46] Muleta H., Fischer L.K., Chang M., Kim N., Leung C.W., Obudulu C., Essel K. (2024). Pediatric produce prescription initiatives in the U.S.: A scoping review. Pediatr. Res..

[47] Owens C.E., Cook M., Reasoner T., McLean A., Webb Girard A. (2024). Engagement in a pilot produce prescription program in rural and urban counties in the Southeast United States. Front. Public Health.

[48] Seligman H.K., Lyles C., Marshall M.B., Prendergast K., Smith M.C., Headings A., Bradshaw G., Rosenmoss S., Waxman E. (2015). A Pilot Food Bank Intervention Featuring Diabetes-Appropriate Food Improved Glycemic Control Among Clients In Three States. Health Aff..

[49] Sharma V., Sharma R. (2024). Food is Medicine Initiative for Mitigating Food Insecurity in the United States. J. Prev. Med. Public Health.

[50] Seligman H.K., Berkowitz S.A. (2019). Aligning Programs and Policies to Support Food Security and Public Health Goals in the United States. Annu. Rev. Public Health.

[51] Hager K., Kummer C., Lewin-Zwerdling A., Li Z. (2024). Food Is Medicine Research Action Plan. https://aspenfood.org/wp-content/uploads/2024/04/Food-is-Medicine-Action-Plan-2024-Final.pdf.

[52] Carpenter A., Kuchera A.M., Krall J.S. (2022). Connecting Families at Risk for Food Insecurity With Nutrition Assistance Through a Clinical-Community Direct Referral Model. J. Nutr. Educ. Behav..

